# An Adaptive Rank Aggregation-Based Ensemble Multi-Filter Feature Selection Method in Software Defect Prediction

**DOI:** 10.3390/e23101274

**Published:** 2021-09-29

**Authors:** Abdullateef O. Balogun, Shuib Basri, Luiz Fernando Capretz, Saipunidzam Mahamad, Abdullahi A. Imam, Malek A. Almomani, Victor E. Adeyemo, Ganesh Kumar

**Affiliations:** 1 Department of Computer and Information Science, Universiti Teknologi PETRONAS, Bandar Seri Iskandar 32610, Malaysia; shuib_basri@utp.edu.my (S.B.); saipunidzam_mahamad@utp.edu.my (S.M.); imam.abubakar@utp.edu.my (A.A.I.); ganesh_17005106@utp.edu.my (G.K.); 2 Department of Computer Science, University of Ilorin, Ilorin 1515, Nigeria; 3 Department of Electrical and Computer Engineering, Western University, London, ON N6A 5B9, Canada; lcapretz@uwo.ca; 4 Department of Software Engineering, The World Islamic Sciences and Education University, Amman 11947, Jordan; malek.almomani@wise.edu.jo; 5 School of Built Environment, Engineering and Computing, Leeds Beckett University, Headingley Campus, Leeds LS6 3QS, UK; v.adeyemo@leedsbeckett.ac.uk

**Keywords:** feature selection, high dimensionality, rank aggregation, software defect prediction

## Abstract

Feature selection is known to be an applicable solution to address the problem of high dimensionality in software defect prediction (SDP). However, choosing an appropriate filter feature selection (FFS) method that will generate and guarantee optimal features in SDP is an open research issue, known as the filter rank selection problem. As a solution, the combination of multiple filter methods can alleviate the filter rank selection problem. In this study, a novel adaptive rank aggregation-based ensemble multi-filter feature selection (AREMFFS) method is proposed to resolve high dimensionality and filter rank selection problems in SDP. Specifically, the proposed AREMFFS method is based on assessing and combining the strengths of individual FFS methods by aggregating multiple rank lists in the generation and subsequent selection of top-ranked features to be used in the SDP process. The efficacy of the proposed AREMFFS method is evaluated with decision tree (DT) and naïve Bayes (NB) models on defect datasets from different repositories with diverse defect granularities. Findings from the experimental results indicated the superiority of AREMFFS over other baseline FFS methods that were evaluated, existing rank aggregation based multi-filter FS methods, and variants of AREMFFS as developed in this study. That is, the proposed AREMFFS method not only had a superior effect on prediction performances of SDP models but also outperformed baseline FS methods and existing rank aggregation based multi-filter FS methods. Therefore, this study recommends the combination of multiple FFS methods to utilize the strength of respective FFS methods and take advantage of filter–filter relationships in selecting optimal features for SDP processes.

## 1. Introduction

Software development lifecycle (SDLC) is a structured mechanism explicitly designed and developed for the creation or development of top-quality software systems. To maintain a timely and efficient software system, the incremental measures contained in SDLC, such as requirement elicitation, software system analysis, software system design, and maintenance, should be closely followed and implemented [1,2]. However, since the stepwise processes in SDLC are performed by human experts, mistakes or errors are unavoidable. Currently, due to the large size and dependencies in modules or components of software systems, these mistakes are prominent and re-occurring. Consequently, if not corrected promptly, these mistakes lead to unstable software systems and finally to software failure. That is, the presence of errors in modules or components of software systems will result in defective and low-quality software systems. In addition, glitches in software systems create frustration for end-users and stakeholders when the failed software system does not function as proposed even after exhausting limited resources (time and effort) [3,4]. Therefore, it is crucial to identify and predict software defects before product release or during the software development phase. Early prediction and identification of faulty modules or components in a software system will allow such modules or components to be spontaneously corrected and the available resources to be used judiciously [5,6].

Software defect prediction (SDP) involves the implementation of machine learning (ML) methods to assess the defectivity of modules or components in a software system. In particular, SDP deploys ML methods on software features that are defined by software metrics to contain defects in software modules or components [7,8,9]. Several studies have proposed and implemented both supervised and unsupervised forms of ML methods for SDP [10,11,12,13,14,15]. Nevertheless, the predictive performance of SDP models is flatly dependent on the quality and inherent nature of the software datasets used for developing such SDP models. Software metrics used to characterize the quality and efficiency of software are directly related to its magnitude and complexities. In other words, broad and scalable software systems will require a variety of software metric mechanisms to produce features that better represent the quality of such software systems [16,17]. Generally, software systems with a significant number of features, due to the accumulation of software metrics, are composed mainly of redundant and irrelevant features which can be defined as a problem of high dimensionality.

Several research findings have shown that the high dimensionality of software metrics adversely impacts the predictive efficiency of SDP models [18,19]. The feature selection (FS) method is recognized by many researchers as an effective way of resolving high dimensionality problems. Principally, these FS methods simply extract useful and critical software features from the original software defect dataset for every SDP operation [20,21,22,23]. The implementation of FS methods will then lead to the formation of a subset of features that contains germane and crucial features from a set of irrelevant and excessive features, thus overcoming the high dimensionality of the dataset. In other words, FS methods select prominent features while ensuring the quality of the dataset. In the end, this solves the high dimensionality problem of software defect datasets [24,25].

FS methods can be categorized into two groups: filter feature selection (FFS) and wrapper feature selection (WFS). FFS methods evaluate features of a dataset using inherent numerical or statistical properties from the dataset. Subsequently, top-ranked features are chosen based on the predefined threshold value. Contrary to FSS, WFS methods evaluate features of a dataset based on its effectiveness in enhancing the performance of underlining classifiers. That is, WFS selects features based on classifier performance. This characteristic renders WFS computationally costly and difficult to implement as it often leads to biased and over-fitting models [26,27,28]. Based on this attribute of WFS, researchers usually prefer FSS methods in SDP [18,29]. Nonetheless, selecting a fitting FFS method for SDP is a problem. This is based on findings from existing studies on the impact of FSS in SDP, which concluded that there is no one best FSS method and that their respective performances depend on selected datasets and classifiers [15,19,21,22]. This observation can be due to incomplete and disjointed feature ranking of FFS methods in SDP. Also, the complex and different implicit operational behaviours of FFS methods are another factor that affects the selection of FSS methods in SDP [18,30]. This problem can be described as the filter rank selection problem in SDP, and a viable solution is to hybridize FFS methods by combining and aggregating the rank lists from each FFS method into a single robust and non-disjoint rank list [31,32].

Therefore, this study proposes a novel adaptive rank aggregation-based ensemble multi-filter FS method with a backtracking function (AREMFFS) for SDP. Specifically, the AREMFFS method is based on evaluating and combining the strengths of individual FFS methods by aggregating multiple rank lists in the generation and subsequent selection of top-ranked features to be used in the SDP process.

The main contributions of this study are as follows:

1. To develop a novel adaptive rank aggregation-based ensemble multi-filter FS method with a backtracking function (AREMFFS).
2. To empirically evaluate and validate the performance of AREMFFS against baseline FFS methods and existing rank aggregation-based FS methods.
3. To validate the application of AREMFFS as a solution for the filter rank selection problem and high dimensionality in SDP.

The remainder of this paper is structured as follows: reviews on existing related works are presented in Section 2; details on proposed AREMFFS and experimental methods are described in Section 3. Experimental results are analysed and discussed in Section 4; and the research is concluded with highlights of future works in Section 5.

## 2. Related Works

Software testing is a vital stage in SDLC as it involves the detection of defects or bugs in the codebase of software systems. The challenge in this stage is to accurately detect the defective source code. Developing an SDP solution is a difficult issue, and numerous approaches have been suggested in the literature.

Early SDP solutions are primarily based on assessing the software requirement specification (SRS) documents for possible flaws. For instance, Smidts, Stutzke [33] developed a software reliability model based on SRS and failure modes. Data from the failure mode(s) are passed into the suggested model as input data. Also, Cortellessa, Singh [34] combined the unified modelling language (UML) of software architecture and a Bayesian framework for SDP. These approaches, unfortunately, do not provide or allow code re-use, which indirectly implies that component failures are independent of one another. Similarly, Gaffney and Davis [35,36] suggested a phase-based model for software reliability. The model is mostly based on defect data discovered after a study of different software development stages. However, this approach is restrictive as it is usually tailored to a particular organization. In another study, Al-Jamimi [37] investigated the use of fuzzy logic in SDP. Specifically, Takagi–Sugeno fuzzy inference engine was used for SDP. Also, Yadav and Yadav [38] successfully implemented a fuzzy logic method for phase-wise SDP. They aimed to provide a generic set of features for SDP. Nonetheless, the decidability issue is a prominent short-fall of fuzzy logic [39].

In recent times, as a result of huge code bases, many ML techniques have been developed for SDP. For instance, Khan, Naseem [40] investigated the performance of several supervised ML techniques for SDP. In a similar study, Naseem, Khan [41] explored the performance tree-based classifiers for SDP. Also, Akimova, Bersenev [42] experimented with the performance of deep learning methods in SDP. Findings from these studies indicated that ML approaches can be utilized for detecting defects in SDP processes. Similarly, evolutionary computation (EC) techniques have been deployed for SDP tasks. In particular, Haouari, Souici-Meslati [43] investigated the performance of artificial immune systems, which are bio-inspired ML techniques based on the mammalian immune paradigms for SDP. Also, Khurma, Alsawalqah [44] proposed an efficient binary variant of moth flame optimization (BMFO) for SDP. Concerning unsupervised ML techniques, Xu, Li [45] investigated the applicability and performance of 40 clustering techniques for SDP. In addition, Marjuni, Adji [46] developed an unsupervised ML technique named signed Laplacian-based spectral classifier for SDP. The unsupervised ML technique is applied when working on unlabelled datasets, unlike supervised ML [14]. However, the performance of an ML technique depends largely on the quality of the datasets used for training such an ML technique [47,48,49].

It is important to highlight that SDP is not the only method that can be used to find defects in software systems. For instance, model checking [50] and static code analysis (e.g., Coverity [51]) can be deployed to find defects in software systems. The duo of model checking and static analysis are conventional methods of finding defects in software systems and are based on fault localization. That is, they use the discrepancies between the inputs of failed and successful software tests to identify problems in the source code. These conventional methods often detect flaws in the present code base only (i.e., the codebase being examined), while SDP warns about future defect-prone regions of a software system.

High dimensionality is a data quality problem that affects the predictive performances of SDP models. In other words, the occurrence of redundant and noisy software features due to the amount and increase in the software metrics deployed for determining the quality of a software system has a negative effect on prediction models in SDP. Findings from existing studies have indicated that FS methods can be used to resolve the high dimensionality problem. As such, several studies have proposed diverse FS methods and investigated the effects of these on the predictive performance of SDP models.

Cynthia, Rasul [52] investigated the impact of FS methods on prediction models in SDP. Specifically, the impact of five FS methods was investigated on selected classifiers. Based on their experimental results, they concluded that FS methods have a significant effect (positive) on the prediction performance of the selected classifiers. However, their study was limited in terms of the scope (number of selected FS methods and datasets evaluated) of the study. Also, Akintola, Balogun [1] conducted a comparative study on filter-based FS methods on heterogeneous prediction models. Principal component analysis (PCA), correlation-based feature selection (CFS), and filtered subset evaluation (FSE) were applied on selected classifiers. They also observed that applying FS methods in SDP is beneficial as it improves the prediction performances of selected classifiers. In another study by Balogun, Basri [20], the authors studied the effect of FS methods on SDP models in terms of applied search methods. They argued that the search method used by an FS method could affect its performance. Eighteen FS methods with four classifiers were considered for experimentation. Observations from their results support the use of FS in SDP. They also posited that the performance of FS methods depends on the datasets and classifiers used in the experiments. That is, there is no one best FS method, and by extension, selecting a suitable FS method to be used in SD becomes a problem. This observation can be denoted as the filter rank selection problem in SDP. Balogun, Basri [21] extended their initial study [20] by conducting a large-scale empirical FS study on SDP. The empirical study was anchored on findings accentuated by Ghotra, McIntosh [53] and Xu, Liu [54] in their respective studies. Findings from their study suggested that the performance of FS methods relies on the choice of datasets and classifiers. Hence, there are no best FS methods. This observation further alludes to the filter rank selection problem of FS methods in SDP.

As a viable solution to the filter rank selection problem, Jia [55], in their study, recommended a hybrid FS method that combines three filter FS methods (chi-squared, information gain, and correlation filter). The proposed hybrid FS method selects features from each rank list based on a pre-determined (TopK) value. It was observed that SDP models based on the proposed hybrid FS method were superior to models with the experimental baseline FS methods. However, it should be noted that the overall ranking of features can be influenced by the distorted ranks of each feature [56]. Moreover, the choice of predetermined TopK features is not always the right approach, as important and relevant features may be overlooked during the feature selection [52]. To address the filter rank selection problem, Wang, Khoshgoftaar [57] studied the ensemble of FS techniques in SDP. Using 18 distinct filter FS techniques, 17 ensemble methods were developed. The ensemble techniques were based on averaging feature rankings from separate rank lists. They reported the advantages of the ensemble methods based on their experimental findings. However, as Jia [55] pointed out, average rank lists of characteristics may be influenced by the skewed rankings of each feature.

Xia, Yan [58] used ReliefF and correlation analysis for feature selection in metric-based SDP. The suggested technique (ReliefF-Lc) simultaneously evaluates correlation and redundancy between modules. According to their research findings, ReliefF-Lc surpasses other studied techniques (IG and REF). Malik, Yining [59] have carried out an empirical comparison study on the usage of an attribute rank approach. The usefulness of principal component analysis (PCA) with the ranker search technique as a filter FS method was specifically explored. They concluded that using PCA with a ranker search technique in the SDP process may enhance the efficacy of classifiers in SDP. Despite the fact that their results cannot be generalised owing to the restricted scope of their research, they do agree with previous SDP studies on the use of FS techniques in SDP.

Iqbal and Aftab [29] created an SDP framework that makes use of multi-filter FS and multi-layer perceptron (MLP). In addition, to solve the intrinsic class imbalance issue, a random over-sampling (ROS) method was included. The suggested multi-filter was created by combining four distinct search methods with correlation feature selection (CFS). Based on their findings, they determined that the multi-filter approach with ROS outperformed the other methods tested.

Similarly, Balogun, Basri [31] addressed the filter rank selection problem in SDP by proposing a rank aggregation-based multi-filter FS method, and in another study, Balogun, Basri [32] conducted an empirical comparative analysis of rank aggregation-based FS methods in SDP. Findings from both studies showed that using rank aggregation methods to aggregate multiple rank lists produced by individual FS methods can address the filter rank selection problem in SDP.

Based on the preceding reviews and findings, this study, therefore, presents an adaptive rank aggregation-based ensemble multi-filter FS method for the SDP process.

## 3. Methodology

Details on the choice of classifiers, FS methods, proposed AREMFFS, experimental procedure, datasets, and evaluation measures are provided in this section.

### 3.1. Classification Algorithms

In this study, decision tree (DT) and naïve Bayes (NB) algorithms are selected and deployed as prediction models. The choice of the duo is due to their respective high prediction performance and ability to work on imbalanced datasets [20,60]. Also, DT and NB are typically not affected by parameter tuning. In addition, DT and NB have been used repeatedly in existing SDP studies. As shown in Table 1, the *subTreeRaising* parameter of DT is set to *True* to allow the pruning of trees by pushing nodes upwards towards the tree’s root, replacing other nodes along the way. Also, the *ConfidenceFactor* is at a 0.25 threshold value and the minimum number of objects on a leaf (MinObj) is set to 2. The essence of these parameters for the DT classifier is to ensure a simpler model with a higher number of nodes (samples) [61]. Concerning the NB classifier, a kernel estimator is used for numeric attributes and the number of decimal places to be used for the output of numbers in the model is set to 2 [62].

### 3.2. Feature Selection Method

Concerning the baseline FS methods, three filter FS methods with diverse computational characteristics were chosen in this study. Specifically, chi-square (CS), Relief (REF), and information gain (IG) were deployed as baseline FS methods. CS is a statistics-based FS method that assesses the degree of independence of an attribute from the class label. REF, an instance-based FS method, samples features of a given dataset and then compares each sampled feature in its respective neighbourhood and thereafter assigns a relevance score to each feature. Finally, the IG method selects features using an entropy mechanism that is based on selecting relevant features by minimizing uncertainties associated with identifying the class label when the value of the feature is unknown. The specific selection of these FS methods (CS, REF, IG) is based on findings from existing studies as they have been regarded to have a high positive effect on prediction performances of SDP models [20,21]. Moreover, these FS methods (CS, REF, IG) are selected to introduce heterogeneity as each of the FS methods have different and unique computational mechanisms. More details on the selected FS methods can be found in [31,32,63,64,65,66]

### 3.3. Adaptive Rank Aggregation-Based Ensemble Multi-Filter Feature Selection (AREMFFS) Method

The proposed AREMFFS method aims to take the computational capabilities of multiple independent filter FS methods into account and incorporate their strengths. The objective of this proposed method is to address filter method selection problems by selecting features and generating a robust rank list from multiple filter FS methods in SDP tasks. AREMMFS can be divided into three stepwise phases: the multi-filter FS phase, the ensemble rank aggregation phase, and the backtracking function phase.

#### 3.3.1. Multi-Filter FS Phase

Individual rank lists from the CS, REF, and IG filter FS methods are constructed from the given datasets as shown in Algorithm 1. The multiple rank lists generated by the independent filter FS methods are mutually exclusive as each filter FS method under consideration has distinct computational features. This is undertaken to guarantee that varied representations of features are chosen. Following that, the generated multiple rank lists are aggregated using rank aggregation functions shown in Table 2. Each rank aggregation function blends the multiple rank lists into a single aggregated rank list by using the significance score assigned to each feature on the individual rank lists.

Specifically, the *Minimum (Min)* and *Maximum (Max)* rank aggregation functions choose features based on the minimum and maximum significance score provided by the aggregated rank list, respectively. The *Arithmetic Mean (Mean)* rank aggregation function aggregates the multiple rank lists into a single aggregated rank list by calculating the arithmetic mean of the significance scores assigned to each element on the individual rank lists. This is done to ensure that each feature from each rank list receives equal representation and consideration. Features with poor significance ratings on the aggregated rank list indicate that they should be ranked low on each rank list and, as such, may be dropped. To select appropriate features, a novel dynamic and automated threshold value dependent on the geometric mean function is added to the aggregated rank list.
**Algorithm 1.** Pseudocode of Proposed AREMFFS.**Input:**n: Total number of features in the datasetN: Total Number of Filter Rank Method = |*CS, REF, IG|*T_1_： Threshold value for optimal features selections = (∏i=1nXi)1n$=\sqrt[{n}]{X_{1}X_{2}X_{3\;}\ldots \;X_{n}}$$T_{2}:\;\log_{2}n$A[]: Aggregators *A* = { *min*
$\{R_{1}\left(a_{1\ldots n}\right),\;R_{2}\left(a_{1\ldots n}\right),\;\ldots \;R_{m}\left(a_{1\ldots n}\right)$*},**max{*$R_{1}\left(a_{1\ldots n}\right),\;R_{2}\left(a_{1\ldots n}\right),\;\ldots \;R_{m}\left(a_{1\ldots n}\right)$*},mean{*(∑i=1mRi(a1…n))×1m*}, }*P[]: Aggregated Features
$P_{t}^{\prime }[]$: Optimal Features Selected from Aggregated Rank List based on T**Output:**$M_{t}^{*}[]$ – Single rank list based on AREMFFS                    // *Multi-Filter Feature Selection Phase*1.  for
i=1 to N { do2.          Generate Rank list R*_n_* for each filter rank method *i*3.        }4. Generate Aggregated Rank list using Aggregator functions:for i=1 to len(A[ ]) { do5.          $P_{t}^{*}\left[i\right]$ // Initialise variable to hold optimal features 6.          $P_{i}=A_{i}$7.          for i =1 to $P_{i}\left[N_{i}\right]\;${ do8.              $\mathrm{if}\;(P_{i}\left[i\right]\leq$ T_1_)9.                    $P_{t}^{*}\left[i\right]\leftarrow P_{i}\left[i\right]$$\;//\;\mathrm{append}\;\mathrm{optimal}\;\mathrm{features}\;\mathrm{from}\;P^{\prime }$ based on T10.                     }11.             }//*Ensemble Rank Aggregation Phase*12.  
$\mathrm{for}\;\mathrm{each}\;\mathrm{feature}f\;\mathrm{in}\;P_{t}^{*}\left[i\right]\;s.t.\;\;i=1,\ldots ,\;A_{n}\;\{\;do\;\;\;\;$// compute the frequency of each feature in the                                aggregated lists13.                           
$\mathrm{if}\;feature\;f_{j}\in \;P_{t}^{*}\left[i\right]$ 14.                               $j=count\;(f_{j\;})$ 15.                            $\mathrm{if}\;\;\left(j\;\geq \;A_{n-1}\right)$ 16.                                $M[\;]\;\leftarrow \;feature\;f_{j}$   //append feature}//select most occurring feature *f* in the aggregated list17.   for i=1 to N{ do18.          Generate Rank list R*_n_* for each filter rank method *i*19.           $\mathrm{for}\;i=1\;\mathrm{to}\;R_{n}$ { do20.                     $R_{i}{}^{\prime }\left[i\right]\;\leftarrow TopK\;features\;of\;R_{n}\;based\;on\;T_{2}\;$21.                   }22.           }*//Backtracking function Phase*23.   
for i=1 to len(M[ ]){ do24.              $\mathrm{for}\;j=1\;\mathrm{to}\;R_{i}{}^{\prime }\left[N\right]$ { do25.                           $\mathrm{if}\;(feature\;f_{i\;}\in \;R_{i}{}^{\prime }\left[j\right]$) 26.                                     $g=count\;\left(f_{i}\right)$
27.                         }28.               $\mathrm{if}\;\left(g\;\geq \;R_{n-1}\right)$29.                  $M_{t}^{*}\left[\;\right]\leftarrow feature\;f_{i}$30.              }31.  $\mathrm{return}\;M_{t}^{*}\left[\;\right]$


The geometric mean of the aggregated significance score is calculated and features with aggregated significance scores less than or equal to the calculated threshold values are chosen. In its calculation, the geometric mean functions take into account feature dependence and the compounding effect. At the end of this phase, aggregated rank lists are generated, based on the *Min*, *Max*, and *Mean* rank aggregation functions. The preference for using *Min*, *Max*, and *Mean* rank aggregation functions is primarily based on the experimental findings reported in [32]. Balogun, Basri [32] deduced that the selected rank aggregation functions (*Min*, *Max*, and *Mean*) can generate a more stable and complete subset of features that best represent studied datasets.

In the next phase of the AREMFFS method, the resulting aggregated rank lists produced by the respective rank aggregation function are ensembled based on the majority voting mechanism to create a single rank list.

#### 3.3.2. Ensemble Rank Aggregation Phase

In this phase, the resulting rank lists generated by the respective rank aggregation functions (*Min*, *Max*, and *Mean*) are ensembled based on a majority voting mechanism to produce a single rank list. As observed, the generated rank lists from each of the rank aggregators are mutually exclusive subsets of features that are considered relevant and important based on each rank aggregator method. Specifically, the ensemble rank aggregation phase of the proposed AREMFFS method selects features that have a frequency value greater than or equal to n−1,  where *n* is the number of aggregated rank lists. In this study, the number of ensembled rank aggregators is *3 (n = 3)*; hence, for a feature to be selected in the ensemble rank aggregation phase, the feature must be a subset of the n−1 rank list generated by the *Min*, *Max*, and *Mean* rank aggregator methods. This approach aims to combine rank lists generated by the respective rank aggregators into a single robust list that best represents each of the combined rank lists. Each rank aggregator has its advantages and disadvantages, and any rank aggregator method deployed should guarantee diversity while increasing the regularity of the FS process, to take advantage of the rank aggregators to boost the prediction performance of SDP models. In addition, the resulting rank list from the ensemble rank aggregation phase is expected to both produce more stable results and minimize the risk of selecting an unstable subset of features. However, there is a need to reduce to some extent the size of the resulting feature set, if large, while maintaining or improving the prediction performance of SDP models. A backtracking function, which is the third phase of the proposed AREMFFS method, is presented in the following sub-section.

#### 3.3.3. Backtracking Function Phase

The last phase of the proposed AREMFFS method is a backtracking function that is applied to the resulting rank list from the ensemble rank aggregation phase. The backtracking function is based on checking the relevance of each feature in the resulting rank list against the initial rank list produced by the individual FFS method. That is, the rank or score of each feature in the resulting rank list is compared with the rank or score features in each of the individual rank lists produced by CS, IG, and REF FS methods. In particular, the backtracking function phase of the proposed AREMFFS method selects features that have a frequency value greater than or equal to n−1, where *n* is the number of FFS methods used in the experiment. Iteratively, the number of FFS methods experimented on is *3 (n = 3)*; hence, for a feature to be selected in the backtracking phase into the optimal subset of features, the feature must be a $\log_{2}N\;$top-ranked feature of at least one n−1 rank list generated by the CS, IG, and REF FS methods. That is, only features from the resulting rank list that are ranked important by at least two of the experimented FFS methods will be selected. This approach aims to confirm the relevancy of features in the optimal resulting rank list and appropriately reduce the size of the resulting feature set while maintaining or improving the prediction performance of SDP models.

### 3.4. Software Defect Datasets

Defect datasets from four publicly available repositories were used for the experiments in this study. Specifically, 25 datasets with varying granularities were selected from PROMISE, NASA, AEEEM, and ReLink repositories. For the NASA repository, the Shepperd, Song [67] version of defect datasets was used. The datasets consist of software features produced by static code metrics. Static code metrics are derived from the source code size and complexity [19,22]. The PROMISE repository contains defect datasets derived from object-oriented metrics and additional information from software modules. This additional information is derived from Apache software [18,22,68]. Concerning the ReLink repository, datasets from this repository are derived from source code information from version control. These datasets were created by Wu, Zhang [69] as linkage data, and have been widely used in existing studies in SDP [62,70,71]. Lastly, the AEEEM datasets contain software features from source code metrics based on change metrics, entropy, and churn of source code metrics [19,22,68,72]. The description of these datasets is presented in Table 3.

### 3.5. Experimental Procedure

**Figure 1:**
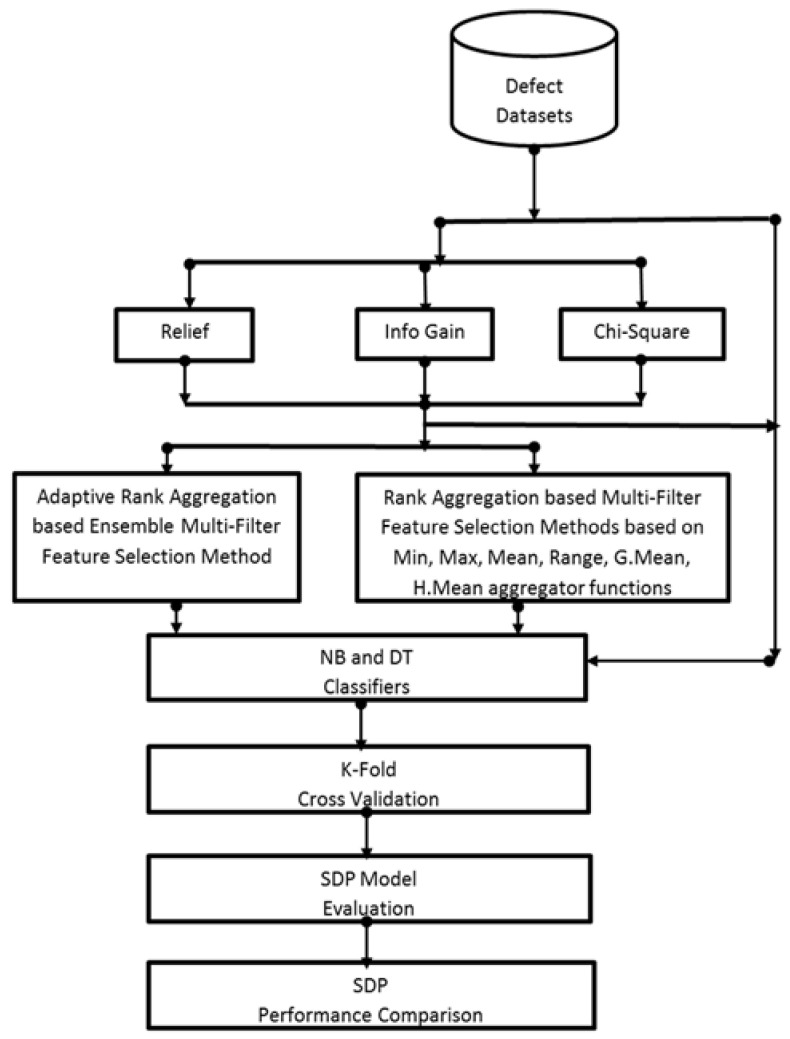
Experimental procedure.

This section presents and analyses the experimental procedure followed in this study as shown in Figure 1.

To evaluate the impact and effectiveness of the proposed AREMFFS on the predictive performance of SDP models, software defect datasets were used to construct SDP models based on the NB and DT classification algorithm. Various scenarios were investigated with non-biased and consistent performance comparative analyses of the resulting SDP models:

- **Scenario A:** In this case, the performance of the proposed AREMFFS method was tested and compared with the baseline FS methods used in this study. The essence of this scenario was to evaluate and validate the performance of the AREMFFS against NoFS, CS, IG, and REF FS methods.
- **Scenario B:** In this case as well, the performance of the proposed AREMFFS method was tested and compared with existing rank aggregation-based multi-filter FS methods as proposed in [31,32]. Findings from this scenario were used to validate the effectiveness of the proposed AREMFFS against Min, Max, Mean, Range, GMean, and HMean rank aggregation-based FS methods.
- **Scenario C:** For this case, the performance of AREMFFS was tested and compared with its variant (REMFFS) as proposed in this study. This allowed a fair comparison and empirically validated the effectiveness of the proposed AREMFF method.

Experimental results and findings based on the aforementioned scenarios were used to answer the following research questions:

- RQ1: How effective is the proposed AREMFFS method compared to baseline FFS methods?
- RQ2: How effective is the proposed AREMFFS method compared to existing rank aggregation-based multi-filter FS methods?

SDP models generated based on the above-listed scenarios were trained and tested using the 10-fold cross-validation (CV) technique. The CV technique mitigates data variability issues that may occur in defect datasets. In addition, the CV technique has been known to produce models with low bias and variance [73,74,75,76,77]. The prediction performances of generated SDP models were assessed using performance evaluation metrics such as accuracy, AUC, and f-measure. The Scott–Knott ESD statistical rank test was used to ascertain the significant differences in the prediction performances of the models used in the experiment. The Weka machine learning library, R lang, and Origin Plot were used for the experimentation [78].

### 3.6. Performance Evaluation Metrics

In terms of performance evaluation, SDP models based on the proposed and other methods were analysed using accuracy, the area under the curve (AUC), and f-measure values, metrics most often used in existing SDP studies to assess the performance of SDP models [6,79].

Accuracy is the amount or proportion of data accurately estimated out of the actual number of data and can be represented as shown in Equation (1):

(1) $$\mathrm{Accuracy}=\frac{\mathrm{TP}+\mathrm{TN}}{\mathrm{TP}+\mathrm{FP}+\mathrm{FN}+\mathrm{TN}}\;\times \;100\%$$

F-Measure is computed based on the harmonic mean of precision and recall values of observed data. Equation (2) presents the formula for calculating the f-measure value:

(2) $$F\;-\mathrm{Measure}=2\times \frac{\mathrm{Precision}\;\times \mathrm{Recall}}{\mathrm{Precision}+\mathrm{Recal}}$$

The area under curve (AUC) signifies the trade-off between true positives and false positives. It demonstrates an aggregate output assessment across all possible classification thresholds:

(3) $$\mathrm{Recall}=\left(\frac{\mathrm{TP}}{\mathrm{TP}+\mathrm{FN}}\right)$$

(4) $$\mathrm{Precision}=\left(\frac{\mathrm{TP}}{\mathrm{TP}+\mathrm{FP}}\right),$$

where TP is true positive (representing accurate prediction), FP is false positive (representing inaccurate prediction), TN is true negative (representing accurate mis-prediction), and FN is false negative (representing inaccurate mis-prediction).

## 4. Results and Discussion

This section presents and discusses experimental results based on the experimental process and procedure described in Section 3.5. Box plots are used to represent the prediction performances (accuracy, AUC, and f-measure values) of NB and DT models based on the various FS methods used. Also, Scott–KnottESD statistical rank tests are used to show significant differences in the prediction performance of developed models.

### 4.1. Experimental Results on Scenario A

In this section, experimental results based on Scenario A as defined in Section 3.5 are presented and discussed. Scenario A is based on assessing and comparing the prediction performances of NB and DT models based on proposed AREMFFS and baseline FS (CS, IG, REF, and NoFS) methods.

**Figure 2:**
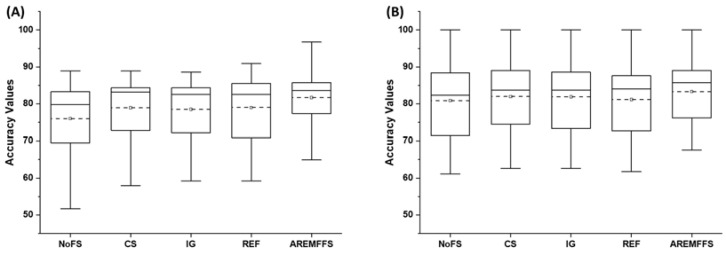
Box-plot representations (accuracy values) of models based on AREMFFS and baseline FFS methods: (**A**) average accuracy values of NB and (**B**) average accuracy values of DT.

**Figure 3:**
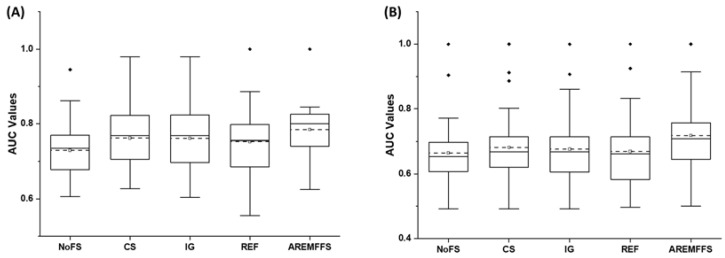
Box-plot representations (AUC values) of models based on AREMFFS and baseline FFS methods: (**A**) average accuracy values of NB and (**B**) average accuracy values of DT.

**Figure 4:**
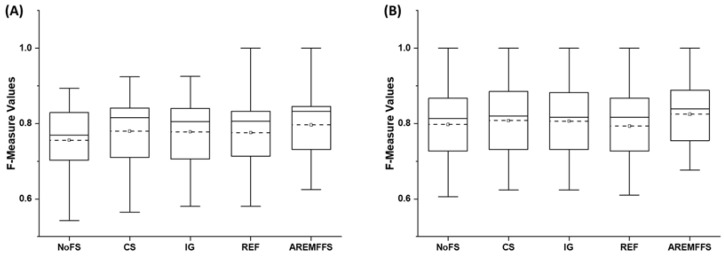
Box-plot representations (F-Measure values) of models based on AREMFFS and baseline FFS methods: (**A**) average accuracy values of NB and (**B**) average accuracy values of DT.

Figure 2, Figure 3 and Figure 4 display box-plot representations of the accuracy, AUC, and f-measure values of NB and DT classifiers with the NoFS method, the baseline FFS (IG, REF, CS) method, and the proposed AREMFFS method. Specifically, the accuracy values of NB and DT classifiers compared with the FS (IG, CS, REF, and AREMFFS) and NoFS methods are presented in Figure 2. The results indicate that NB and DT had good accuracy values on the software defect dataset. Nonetheless, the increased deployment of baseline FFS methods (IG, CS, and REF) further enhanced the accuracy values of NB and DT classifiers. This can be seen in their respective average accuracy values as depicted in Figure 2. Particularly, NB and DT classifiers with the NoFS method had average accuracy values of 76% and 80.89%, respectively. Concerning baseline FFS methods, CS with NB and DT classifiers recorded average accuracy values of 78.93% and 81.97%, which indicated increments of +3.85% and +1.34%, respectively. Identical occurrences were realized in models with IG (NB: 78.51%, DT: 81.9%) and REF (NB: 78.99%, DT: 81.17%) FS methods, with increments of average accuracy values (IG: (+3.32%, +1.25%) and DT: (+3.93%, +0.3%)), respectively. However, models based on AREMFFS with NB and DT classifiers had superior average accuracy values over the NB and DT models with baseline FFS (NoFS, CS, IG, REF) methods. As presented in Table 4, concerning models based on the NB classifier, AREMFFS had increments of +7.46%, +3.47%, +4.02%, and +3.39% in the average accuracy values over models based on the NoFS, CS, IG, and REF methods, respectively. Also, concerning models based on the DT classifier as shown in Table 5, AREMFFS had increments of +3%, +1.63%, +1.72%, and +2.64% in average accuracy values over models based on the NoFS, CS, IG, and REF methods, respectively. These results showed that, concerning accuracy values, models based on AREMFFS outperformed models based on baseline FSS (CS, IG, REF) methods. That is, AREMFFS had a superior positive impact on the prediction accuracy values of NB and DT models over the CS, IG, and REF FS methods.

In terms of AUC values, Figure 3 displays box-plot representations of models based on NB and DT classifiers with baseline FFS and proposed AREMFFS methods. Similar to observations on accuracy values, NB and DT models based on baseline FFS (CS, IG, and REF) had superior AUC values when compared with NB and DT models with the NoFS method. Specifically, CS had increments of +4.4% and +2.55% in AUC values for models based on NB (0.762) and DT (0.682) over the NoFS (NB: 0.73, DT: 0.665) method. Correspondingly, NB and DT models based on IG had increments of +4.25% and +1.65% in average AUC values. Also, models based on REF recorded increments of +3.15% and +0.6% in average AUC values for NB and DT classifiers, respectively, when compared with models with the NoFS method. Nonetheless, similar to the observations on accuracy values, models based on AREMFFS had superior average AUC values over models with baseline FFS (NoFS, CS, IG, REF) methods. As shown in Table 4, concerning models based on the NB classifier, AREMFFS had increments of +7.4%, +2.88%, +3.02%, and +4.12% in average AUC values over models based on the NoFS, CS, IG, and REF methods, respectively. A similar case is observed with models based on the DT classifier as shown in Table 5. AREMFFS had increments of +8.72%, +6.01%, +6.95%, and +8.07% in average AUC values over models based on the NoFS, CS, IG, and REF methods, respectively.

Also, Figure 4 presents the f-measure values for NB and DT models based on the baseline FFS and proposed AREMFFS methods. Models based on the NB classifier with the CS (0.779), IG (0.778), and REF (0.776) methods recorded average f-measure value increases of +3.04%, +2.91%, and +2.64%, respectively, over the NB model with the NoFS method. As for the DT models, IG and CS recorded increments of +1.38% and +1.13% in average f-measure values, but DT models with the REF method performed poorly with a −0.5% decrease of the f-measure value. Summarily, it can be observed that models based on FS methods had better prediction performance than models with the NoFS method. However, models based on AREMFFS with NB and DT classifiers recorded better average f-measure values over NB and DT models with baseline FFS (CS, IG, REF) methods. From Table 4, the NB model based on AREMFFS had increments of +5.42%, +2.31%, +2.44%, and +2.71% in average f-measure values over models based on the NoFS, CS, IG, and REF methods, respectively. Also, the DT model with AREMFFS had increments of +3.51%, +2.1%, +2.36%, and +4.04% in average f-measure values over models based on the NoFS, CS, IG, and REF methods, respectively. These results further indicate the superiority of models (NB and DT) based on AREMFFS over models based on baseline FSS (NoFS, CS, IG, REF) methods.

Summarily, experimental results, as displayed in Figure 2, Figure 3 and Figure 4, showed that the deployment of FS methods in SDP further enhances the prediction performances of SDP models. This finding is supported by observations in existing studies where FS methods are applied in SDP [19,20,21,22]. Nonetheless, it was also observed that the effect of FS methods varies and depends on the classifiers selected in this study. Also, there are no clear-cut differences in the performances of each of the FFS (CS, IG, and REF) methods, even though the selected FFS methods have different underlying computational characteristics. Thus, the selection of an appropriate FFS method to be used in SDP processes becomes a problem that can be termed a filter rank selection problem. This observation from the experimental results strengthens the aim of our study, which proposes a rank aggregation-based multi-filter FS method for SDP. As shown in Figure 2, Figure 3 and Figure 4, the proposed AREMFFS method not only had a superior positive impact on NB and DT models, but also had a more positive impact than the individual CS, IG, and REF FS methods. Particularly, Table 4 and Table 5 present the prediction performances (average accuracy, average AUC, and average f-measure) of NB and DT models with the proposed AREMFFS methods and the experimented baseline FFS (NoFS, CS, IG, REF) methods, respectively.

**Figure 5:**
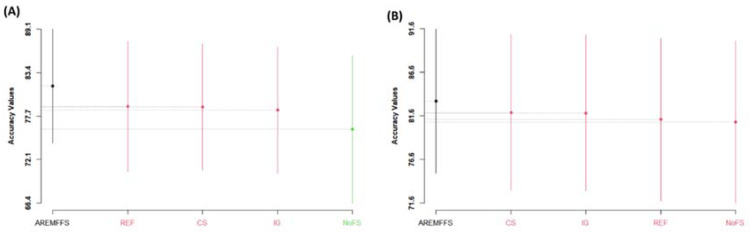
Scott–KnottESD rank test result of models based on AREMFFS and baseline FFS methods: (**A**) average accuracy values of NB and (**B**) average accuracy values of DT.

**Figure 6:**
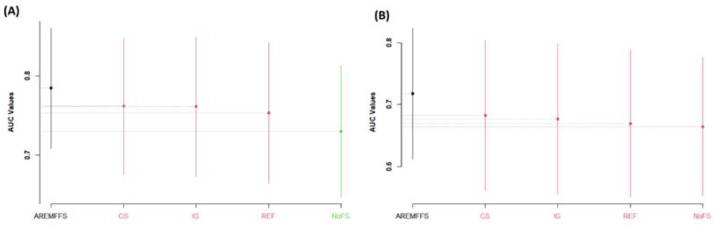
Scott–KnottESD rank test result of models based on AREMFFS and baseline FFS methods: (**A**) average AUC values of NB and (**B**) average AUC values of DT.

**Figure 7:**
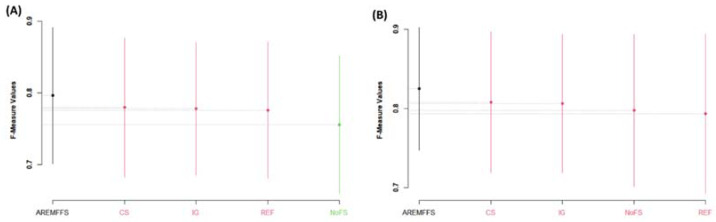
Scott–KnottESD rank test result of models based on AREMFFS and baseline FFS methods: (**A**) average F-Measure values of NB (**B**) average f-measure values of DT.

Figure 5, Figure 6 and Figure 7 present statistical rank tests of the models (NB and DT) tested based on accuracy, AUC, and f-measure values, respectively. Specifically, the Scott–KnottESD statistical rank test, a mean comparison approach that uses hierarchical clustering to separate mean values into statistically distinct clusters with non-negligible mean differences, was conducted [62,80] to show significant statistical differences in the mean values of methods and results used. As depicted in Figure 5, Figure 6 and Figure 7, models with different colours show that there are statistically significant differences amongst their values; hence, they are grouped into a different category. Similarly, models with the same colour indicate that there are no statistically significant differences in their values.

As presented in Figure 5, there are statistically significant differences in the average accuracy values of NB and DT models with the proposed AREMFFS method when compared with other FS methods. In particular, for NB models, AREMFFS ranks highest (first), followed by REF, CS, and IG, which are in the same category, while the NoFS method ranks last. In the case of DT models, AREMFFS still ranks highest followed by other FS methods (CS, IG, REF, and NoFS). It should be noted that the arrangements of models from the statistical rank test are vital as models that appear first (from left to right) are superior to the other models, irrespective of their category. This observation indicates that models based on AREMFFS have superior accuracy values over models based on CS, IG, REF, and NoFS methods. Also, similar observations were recorded from statistical rank tests based on AUC values. Figure 6 presents the Scott–KnottESD statistical rank tests based on AUC values, and there too models based on AREMFFS rank highest. In terms of NB models, AREMFFS ranks highest followed by CS, IG, and REF, which are in the same category, while the NoFS method ranks last. As for DT models, AREMFFS still ranks highest, followed by CS, IG, REF, and NoFS FS methods. Lastly, statistical rank tests based on f-measure values, as shown in Figure 7, followed the same pattern as that of accuracy and AUC values, with models based on AREMFFS being statistically superior to the other experimented baseline FS methods. A summary of the Scott–KnottESD statistical rank tests of the proposed AREMFFS and baseline FS methods with NB and DT classifiers is presented in Table 6.

In summary, from the experimental and statistical test results, the proposed AREMFFS method recorded a superior positive impact on the prediction performances of SDP models (NB and DT) in comparison with individual FSS (CS, IG, REF, and NoFS) methods on the defect datasets that were studied.

### 4.2. Experimental Results on Scenario B

This section presents and discusses experimental results based on Scenario B (see Section 3.5). Scenario B is defined by evaluating and comparing the prediction performances of NB and DT models based on the proposed AREMFFS method and the existing (Min, Max, Mean, Range, GMean, HMean) rank aggregation-based multi-filter FS methods.

**Figure 8:**
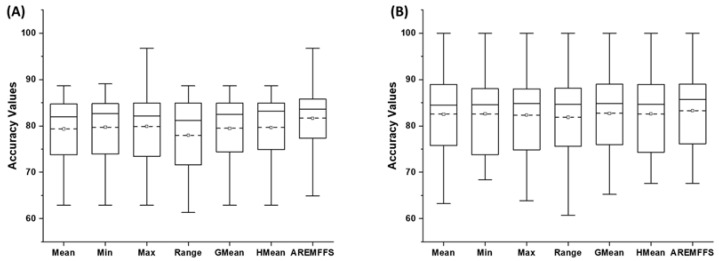
Box-plot representations (accuracy values) of models based on AREMFFS and existing rank aggregation-based multi-filter FS methods: (**A**) average accuracy values of NB and (**B**) average accuracy values of DT.

**Figure 9:**
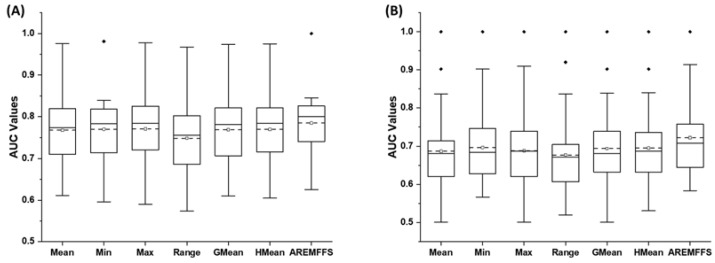
Box-plot representations (AUC values) of models based on AREMFFS and existing rank aggregation-based multi-filter FS methods: (**A**) average accuracy values of NB and (**B**) average accuracy values of DT.

**Figure 10:**
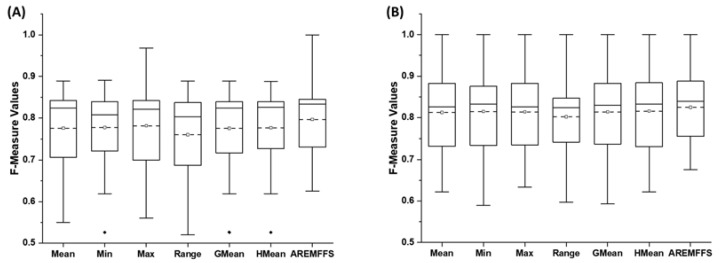
Box-plot representations (f-measure values) of models based on AREMFFS and existing rank aggregation-based multi-filter FS methods: (**A**) average accuracy values of NB and (**B**) average accuracy values of DT.

Figure 8, Figure 9 and Figure 10 show box-plot representations of the accuracy, AUC, and f-measure values of NB and DT classifiers with proposed AREMFFS and existing rank aggregation-based multi-filter FS methods. In particular, Figure 8 presents the accuracy values of NB and DT models with AREMFFS and existing rank aggregation-based multi-filter FS methods. It can be observed that models based on AREMFFS (NB: 81.67,% DT: 83.31%) had superior average accuracy values compared to existing Min (NB: 79.72%, DT: 82.60%), Max (NB: 79.88%, DT: 82.34%), Mean (NB: 79.36%, DT: 82.53%), Range (NB: 77.99%, DT: 81.87%), GMean (NB: 79.48%, DT: 82.70%), and HMean (NB: 79.66%, DT: 82.61%) rank aggregation-based multi-filter FS methods. Specifically, based on NB models, AREMFFS had increments of +2.91%, +2.45%, +2.24%, +4.72%, +2.76%, and +2.52% in average accuracy value over the existing Mean, Min, Max, Range, GMean, and HMean rank aggregation-based multi-filter FS methods. Likewise for DT models, AREMFFS had increments of +0.95%, +0.86%, +1.18%, +1.76%, +0.74%, and +0.85% in average accuracy value over the existing Min, Max, Mean, Range, GMean, and HMean rank aggregation-based multi-filter FS methods. As observed, the experimental results indicate that models based on AREMFFS outperformed models based on existing rank aggregation-based multi-filter FS methods on accuracy values. In other words, AREMFFS had a superior positive impact on the prediction accuracy values of NB and DT models over the Min, Max, Mean, Range, GMean, and HMean rank aggregation-based multi-filter FS methods.

Concerning AUC values, Figure 9 presents box-plot representations of models based on NB and DT classifiers with the proposed AREMFFS and existing rank aggregation based multi-filter FS methods. Similar to observations on accuracy values, models based on AREMFFS (NB: 0.784, DT: 0.723) had superior average AUC values over models with existing Min (NB: 0.769 DT: 0.697), Max (NB: 0.770, DT: 0.688), Mean (NB: 0.767, DT: 0.687), Range (NB: 0.748, DT: 0.677), GMean (NB: 0.768, DT: 0.694), and HMean (NB: 0.769, DT: 0.696) rank aggregation-based multi-filter FS methods. As presented in Table 7, concerning models based on NB classifier, AREMFFS had increments of +2.22%, +1.95%, +1.82%, +4.81, +2.08%, and +1.95% in average AUC values over models based on Mean, Min, Max, Range, GMean, and HMean rank aggregation-based multi-filter FS methods, respectively. A similar case is observed with models based on the DT classifier as depicted in Table 8. AREMFFS had increments of +5.24%, +3.73%, +5.09%, +6.79%, +4.18%, and +3.88% in average AUC values over models based on Mean, Min, Max, Range, GMean, and HMean rank aggregation-based multi-filter FS methods, respectively. As observed, the experimental results indicated that models based on AREMFFS outperformed models based on existing rank aggregation-based multi-filter FS methods on accuracy values. In other words, AREMFFS had a superior positive impact on the prediction accuracy values of NB and DT models over Min, Max, Mean, Range, GMean, and HMean rank aggregation-based multi-filter FS methods.

Furthermore, concerning f-measure values, Figure 10 presents box-plot representations of models with NB and DT classifiers with proposed AREMFFS and existing rank aggregation based multi-filter FS methods. Models based on AREMFFS (NB: 0.797, DT: 0.825) had superior average f-measure values over models with existing Min (NB: 0.777, DT: 0.815), Max (NB: 0.781, DT: 0.814), Mean (NB: 0.775, DT: 0.813), Range (NB: 0.789, DT: 0.802), GMean (NB: 0.775, DT: 0.814), and HMean (NB: 0.776, DT: 0.815) rank aggregation-based multi-filter FS methods. Specifically, based on the NB classifier, AREMFFS had increments of +2.84%, +2.57%, +2.05%, +5.00, +2.84%, and +2.71% in average AUC values over models based on Mean, Min, Max, Range, GMean, and HMean rank aggregation-based multi-filter FS methods, respectively. Also, based on the DT classifier, AREMFFS had increments of +1.48%, +1.23%, +1.35%, +2.87%, +1.35%, and +1.23% in average AUC values over models based on Mean, Min, Max, Range, GMean, and HMean rank aggregation-based multi-filter FS methods, respectively. As observed, the experimental results indicated that models based on AREMFFS outperformed models based on existing rank aggregation-based multi-filter FS methods on f-measure values. In other words, AREMFFS had a superior positive impact on the f-measure values of NB and DT models over Min, Max, Mean, Range, GMean, and HMean rank aggregation-based multi-filter FS methods.

In summary, the findings from the experimental results, as shown in Figure 8, Figure 9 and Figure 10, indicate the superiority of the proposed AREMFFS over existing rank aggregation-based multi-filter FS methods. That is, NB and DT models based on AREMFFS outperformed NB and DT models based on existing Mean, Min, Max, Range, GMean, and HMean rank aggregation-based multi-filter FS methods. The superior performance of the proposed AREMFFS can be attributed to a combination of the robust strategy it deploys for aggregating multiple rank lists based on majority voting, and its backtracking ability which further removes irrelevant features from the generated optimal feature list.

**Figure 11:**
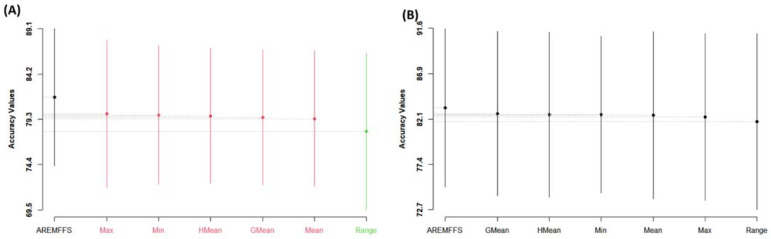
Scott–KnottESD rank test result of models based on AREMFFS and existing rank aggregation-based multi-filter FS methods: (**A**) average accuracy values of NB and (**B**) average accuracy values of DT.

**Figure 12:**
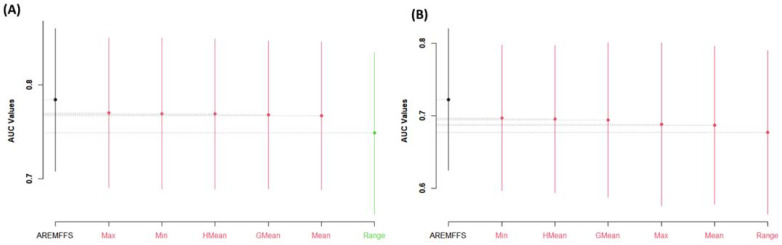
Scott–KnottESD rank test result of models based on AREMFFS and existing rank aggregation-based multi-filter FS methods: (**A**) average AUC values of NB and (**B**) average AUC values of DT.

**Figure 13:**
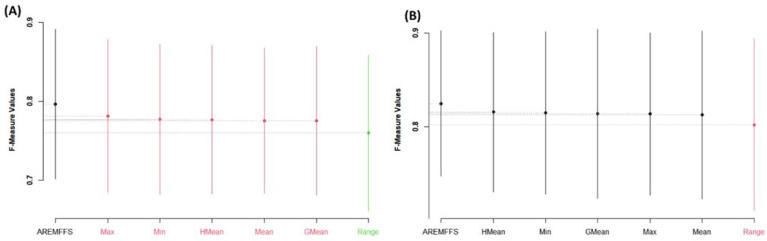
Scott–KnottESD Rank test result of models based on AREMFFS and existing rank aggregation-based multi-filter FS methods. (**A**) average f-measure values of NB and (**B**) average f-measure values of DT.

For further analyses, the performance of the proposed AREMFFS and the existing experimented rank aggregation-based multi-filter FS methods were subjected to Scott–KnottESD statistical rank tests to determine the statistically significant differences in their respective performances. Figure 11, Figure 12 and Figure 13 present statistical rank tests of the proposed AREMFFS and existing rank aggregation-based multi-filter FS methods on NB and DT classifiers based on accuracy, AUC, and f-measure values, respectively. As depicted in Figure 11A, it can be observed that there are statistically significant differences in the average accuracy values of NB models using the proposed AREMFFS when compared with NB models based on existing rank aggregation-based multi-filter FS methods.

Specifically, AREMFFS ranks highest (first), followed by Max, Min, HMean, GMean, and Mean, which are in the same category, while the Range aggregation method ranks last. In the case of DT models as presented in Figure 11B, although there is no significant statistical difference in the prediction accuracy values, AREMFFS still ranks highest, followed by the GMean, HMean, Min, Mean, Max, and Range aggregation methods. In this case, the order of superiority/arrangements (left to right) of the models from the statistical test was considered and AREMFFS appeared first. These findings indicate that models based on AREMFFS have superior accuracy values over models based on Min, Max, Mean, Range, GMean, and HMean rank aggregation-based multi-filter FS methods. Also, similar observations were recorded from statistical rank tests based on AUC values. Figure 12 presents the Scott–KnottESD statistical rank tests based on AUC values, and models based on AREMFFS rank highest. In terms of NB models, AREMFFS ranks highest followed by Max, Min, HMean, GMean, and Mean, which are in the same category, while the Range rank aggregation method ranks last. A similar situation can be observed in the case of DT models as AREMFFS ranks highest followed by the Min, HMean, GMean, Max, Mean, and Range rank aggregation methods. In addition, statistical rank tests based on f-measure values, as presented in Figure 13, indicated similar findings to those of the accuracy and AUC values with Min, Max, Mean, Range, GMean, and HMean rank aggregation-based multi-filter FS methods. Table 9 summarizes and presents the Scott–KnottESD statistical rank tests of proposed AREMFFS and existing rank aggregation-based multi-filter FS methods with NB and DT classifiers.

In summary, based on the experimental and statistical test results, the proposed AREMFFS method recorded a superior positive impact on the prediction performances of SDP models (NB and DT) over existing rank aggregation-based multi-filter FS (Min, Max, Mean, Range, GMean, HMean) methods on the defect datasets studied.

### 4.3. Experimental Results on Scenario C

In this section, experimental results based on Scenario C (See Section 3.5) are presented and discussed. Scenario C is based on assessing and comparing the prediction performances of NB and DT models based on the proposed AREMFFS and its variant (REMFFS: rank aggregation-based ensemble multi-filter feature selection) as proposed in this study. The REMFFS method is based on the same working principle as AREMFFS but without the backtracking function included. The results of this analysis will allow for a fair comparison between the two and empirically validate the effectiveness of the proposed AREMFF method.

**Figure 14:**
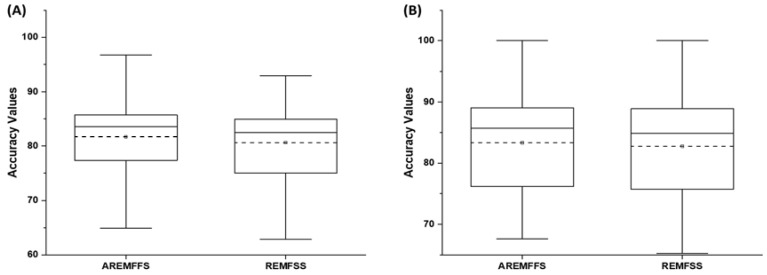
Box-plot representations (accuracy values) of models based on AREMFFS and REMFFS: (**A**) average accuracy values of NB and (**B**) average accuracy values of DT.

**Figure 15:**
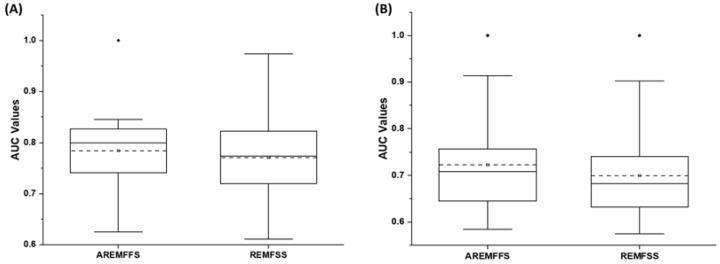
Box-plot representations (AUC values) of models based on AREMFFS and REMFFS: (**A**) average accuracy values of NB and (**B**) average accuracy values of DT.

**Figure 16:**
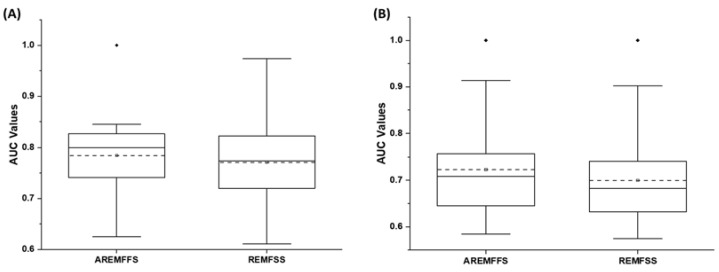
Box-plot representations (f-measure values) of models based on AREMFFS and REMFFS: (**A**) average accuracy values of NB and (**B**) average accuracy values of DT.

Figure 14, Figure 15 and Figure 16 present box-plot representations of the accuracy, AUC, and f-measure values of NB and DT classifiers with the proposed AREMFFS and REMFFS methods. Correspondingly, Figure 14 presents the accuracy values of NB and DT models with the AREMFFS and REMFFS methods. It can be observed that models based on AREMFFS (NB: 81.67%, DT: 83.31%) had superior average accuracy values when compared with the REMFFS (NB: 80.62%, DT: 82.75%) methods. In particular, based on NB and DT models, AREMFFS had increments of +1.3% and +0.67% in average accuracy values, respectively, over the REMFFS method. As observed, the experimental results indicated that models based on AREMFFS outperformed models based on REMFFS on accuracy values. That is, AREMFFS had a superior positive impact on the prediction accuracy values of NB and DT models over the REMFFS method.

Regarding AUC values, Figure 15 shows box-plot representations of models based on NB and DT classifiers with the proposed AREMFFS and REMFFS methods. As presented in Table 10 and Table 11, models based on AREMFFS (NB: 0.784, DT: 0.723) had superior average AUC values over models with REMFFS (NB: 0.771, DT: 0.699). Specifically, NB and DT models with AREMFFS had increments of +1.69% and +3.43% in average AUC values, respectively, over models based on the REMFFS method. As observed, the experimental results indicated that models based on AREMFFS outperformed models based on REMFFS. In other words, AREMFFS had a superior positive impact on the AUC values of NB and DT models over the REMFFS method. Also, in terms of f-measure values, Figure 16 presents box-plot representations of models with NB and DT classifiers with proposed AREMFFS and REMFFS methods. Models based on AREMFFS (NB: 0.797, DT: 0.825) had superior average f-measure values over models with the REMFFS (NB: 0.778, DT: 0.813) method. In particular, NB and DT models with AREMFFS had increments of +2.44% and +1.48% in average f-measure values, respectively, over models based on the REMFFS method. Similarly, the experimental results showed the superiority of models based on the proposed AREMFFS as it outperformed models based on REMFFS on f-measure values. That is, AREMFFS had a superior positive impact on the f-measure values of NB and DT models over the REMFFS method.

**Figure 17:**
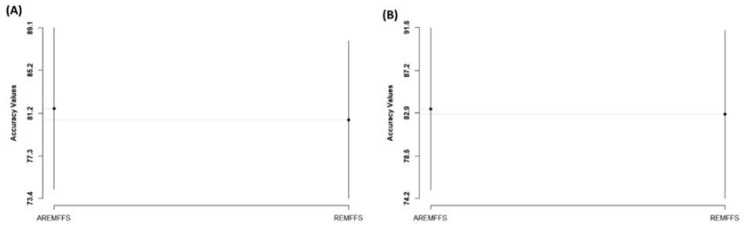
Scott–KnottESD rank test result of models based on AREMFFS and REMFFS: (**A**) average accuracy values of NB and (**B**) average accuracy values of DT.

**Figure 18:**
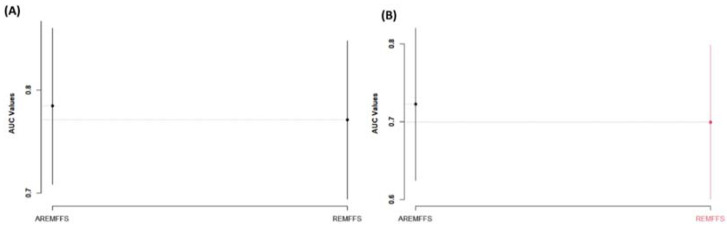
Scott–KnottESD rank test result of models based on AREMFFS and REMFFS: (**A**) average AUC values of NB and (**B**) average AUC values of DT.

**Figure 19:**
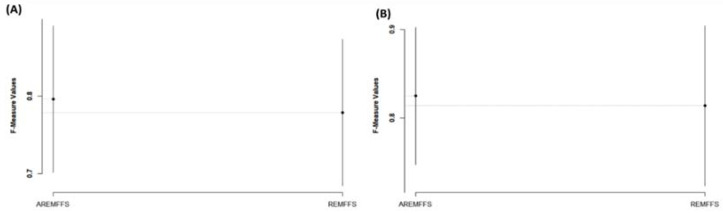
Scott–KnottESD rank test result of models based on AREMFFS and REMFFS: (**A**) average f-measure values of NB and (**B**) average f-measure values of DT.

Based on the preceding experimental results as presented in Figure 14, Figure 15 and Figure 16, the superiority of the proposed AREMFFS over REMFFS can be observed. Correspondingly, the observed superior performance of the proposed AREMFFS over REMFFS can be attributed to its backtracking ability to further remove irrelevant features from the generated optimal feature list. As established, the removal of irrelevant features will further improve the performance of the proposed AREMFFS method. However, it should be noted that REMFFS, which is a variant of AREMFFS, generated a good and competitive prediction performance. Figure 17, Figure 18 and Figure 19 further analysed the performance of AREMFFS and REMFFS methods statistically. In other words, the Scott–KnottESD statistical rank test was used to determine the statistically significant differences in their respective performances based on accuracy, AUC, and f-measure values, respectively.

As presented in Figure 17, it can be observed that there are no statistically significant differences in the average accuracy values of NB and DT models with the proposed AREMFFS and REMFFS methods. Nonetheless, AREMFFS still ranks higher than the REMFFS method when the order of superiority/arrangements (left to right) of the models from the statistical test were considered; that is, AREMFFS appeared first.

From Figure 19, similar observations were recorded from statistical rank tests based on f-measure values, as there is no statistically significant difference in the average f-measure values of models based on the two methods (AREMFFS and REMFFS), although AREMFFS is better. The case is slightly different for Scott–KnottESD statistical rank tests based on AUC values as shown in Figure 18. In terms of NB models (Figure 18A), AREMFFS and REMFFS fall into the same grouping. That is, the difference between their respective AUC values is statistically insignificant. However, for DT models (Figure 18B), AREMFFS is statistically superior to REMFFS, as there is a significant difference in their AUC values. In addition, a summary of the statistical test analyses on the performance of AREMFFS and REMFFS methods is presented in Table 12. These findings indicate that models based on AREMFFS have superior performance over models based on the REMFFS method.

Based on the experimental and statistical test results, the proposed AREMFFS method recorded a superior positive impact on the prediction performances of SDP models (NB and DT) over its variant (REMFFS method) for the defect datasets that were studied.

In summary, from the experimental results and statistical test analyses, the proposed AREMFFS method had a superior positive effect on the prediction performances of SDP models (NB and DT) compared to the individual filter FS methods (CS, IG, REF, and NoFS), existing rank aggregation based multi-filter FS methods (Min, Max, Mean, Range, GMean, and HMean), and its variant (REMFFS) on the defect datasets studied. These findings, therefore, answer RQ1 and RQ2 (see Section 3.6) as presented in Table 13. Furthermore, the efficacy of AREMFFS solves the filter rank selection problem in SDP by integrating the power of individual filter FS methods. As a result, combining filter (multi-filter) methods is suggested as a viable choice for harnessing the power of the respective FFS and the strengths of filter–filter relationships in selecting germane features for FS methods as conducted in this report

## 5. Conclusions

This study focuses on resolving high dimensionality and filter rank selection problems in software defect prediction by proposing a novel AREMFFS method. Selecting an applicable and pertinent filter FS method to be used in SDP is often a problem as the efficacy of these filter FS methods varies. As such, AREMFFS is proposed and designed to combine multiple rank lists generated by different filter FS methods into a single robust rank list. Moreover, a geometric mean function and a backtracking function are used to automatically select top-ranked features and reduce the number of features on the aggregated list. For performance evaluation and validation, NB and DT models were developed using features generated by AREMFFS, selected baseline filter FS methods (IG, CS, REF, and NoFS methods), existing rank aggregation based multi-filter FS methods (Min, Max, Mean, Range, GMean, and HMean), and variants of AREMFFS (REMFFS) for defect datasets with different granularity. Findings from the experimental results indicated the efficacy and superiority of the proposed AREMFFS method as it recorded a better positive impact on the prediction performances of NB and DT classifiers than the other experimented FS methods in most cases.

In particular, the proposed ARMFFS was able to generate a more stable and complete subset of features that best represented the datasets studied. These findings, therefore, support the combination and aggregation of rank lists from multiple filter FS methods as a feasible solution to primarily high dimensionality and filter rank selection problems in SDP. In a wider sense, the results and findings from this research can be used by experts and researchers in SDP and other applicable research domains that require FS methods to address high dimensionality and filter rank selection problems.

Furthermore, in this study, there is a trade-off of the computational time for prediction performance. That is, the computational time is not used as an assessment metric. This is because in most cases, ensemble methods have been reported to record more computational time than single methods [81]. Moreover, the overhead cost of a software defect misprediction could have dire consequences.

As it is a limitation of this study, we plan to investigate and broaden the scope of this study in the future by analysing other ensemble configurations of the FS method with more prediction models. Application of EC techniques for selection of features and reduction in computational time of ensemble methods will be explored. Furthermore, the effect of threshold values on FFS efficacies is worth examining, as the appropriate threshold value is dependent on the dataset used.

## Figures and Tables

**Table 1:** Classification algorithms.

Classification Algorithms	Parameter Settings
Decision Tree (DT)	ConfidenceFactor = 0.25; MinObj = 2; subTreeRaising = True
Naïve Bayes (NB)	NumDecimalPlaces = 2; UseEstimator = True

**Table 2:** Rank aggregation methods.

Aggregators	Formula	Description
Min ()	*min*{ $R_{1}\left(a_{1\ldots n}\right),\;R_{2}\left(a_{1\ldots n}\right),\;\ldots \;R_{m}\left(a_{1\ldots n}\right)$}	Selects the *minimum* of the relevance scores produced by the aggregated rank list
Max ()	*max*{ $R_{1}\left(a_{1\ldots n}\right),\;R_{2}\left(a_{1\ldots n}\right),\;\ldots \;R_{m}\left(a_{1\ldots n}\right)\}$	Selects the *maximum* of the relevance scores produced by the aggregated rank list
Mean ()	*mean{* (∑i=1mRi(a1…n))×1m	Selects the *mean* of the relevance scores produced by the aggregated rank list

**Table 3:** Description of software defect datasets.

Datasets	Number of Features	Number of Modules
EQ	62	324
JDT	62	997
ML	62	1862
PDE	62	1497
CM1	38	327
KC1	22	1162
KC2	22	522
KC3	40	194
MW1	38	250
PC1	38	679
PC3	38	1077
PC4	38	1287
PC5	39	1711
ANT	22	292
CAMEL	21	339
JEDIT	22	312
REDKITOR	21	176
TOMCAT	22	852
VELOCITY	21	196
XALAN	22	797
SAFE	27	56
ZXING	27	399
APACHE	27	194
ECLIPSE	19	1065
SWT	18	1485

**Table 4:** Models based on the NB classifier with proposed AREMFFS and baseline FFS methods.

Models	Average Accuracy (%)	Average AUC	Average F-Measure
NoFS	76.00	0.730	0.756
CS	78.93	0.762	0.779
IG	78.51	0.761	0.778
REF	78.99	0.753	0.776
AREMFFS	81.67	0.784	0.797

**Table 5:** Models based on the DT classifier with proposed AREMFFS and baseline FFS methods.

Models	Average Accuracy (%)	Average AUC	Average F-Measure
NoFS	80.89	0.665	0.797
CS	81.97	0.682	0.808
IG	81.90	0.676	0.806
REF	81.17	0.669	0.793
AREMFFS	83.31	0.723	0.825

**Table 6:** Summary of the Scott–Knott rank test of proposed AREMFFS and baseline FS methods.

Statistical Rank	Average Accuracy		Average AUC		Average F-Measure	
	NB	DT	NB	DT	NB	DT
1	AREMFFS	AREMFFS	AREMFFS	AREMFFS	AREMFFS	AREMFFS
2	REF, CS, IG	CS, IG, REF, NoFS	CS, IG, REF	CS, IG, REF, NoFS	CS, IG, REF	CS, IG, REF, NoFS
3	NoFS	-	NoFS	-	NoFS	-

**Table 7:** Models based on the NB classifier with proposed AREMFFS and existing rank aggregation-based multi-filter FS methods.

Models	Average Accuracy (%)	Average AUC	Average F-Measure
Mean	79.36	0.767	0.775
Min	79.72	0.769	0.777
Max	79.88	0.770	0.781
Range	77.99	0.748	0.759
GMean	79.48	0.768	0.775
HMean	79.66	0.769	0.776
AREMFFS	81.67	0.784	0.797

**Table 8:** Models based on the DT classifier with proposed AREMFFS and existing rank aggregation-based multi-filter FS methods.

Models	Average Accuracy (%)	Average AUC	Average F-Measure
Mean	82.53	0.687	0.813
Min	82.60	0.697	0.815
Max	82.34	0.688	0.814
Range	81.87	0.677	0.802
GMean	82.70	0.694	0.814
HMean	82.61	0.696	0.815
AREMFFS	83.31	0.723	0.825

**Table 9:** Summary of Scott–KnottESD statistical rank tests of proposed AREMFFS and existing rank aggregation-based multi-filter FS methods.

Statistical Rank	Average Accuracy		Average AUC		Average F-Measure	
	NB	DT	NB	DT	NB	DT
1	AREMFFS	AREMFFS, GMean, HMean, Min, Mean, Max, Range	AREMFFS	AREMFFS	AREMFFS	AREMFFS, HMean, Min, GMean, Max, Mean,
2	Max, Min, HMean, GMean, Mean	-	Max, Min, HMean, GMean, Mean	Min, HMean, GMean, Max, Mean, Range	Max, Min, HMean, Mean, GMean	Range
3	Range	-	Range	-	Range	-

**Table 10:** Models based on the NB classifier with the proposed AREMFFS and REMFFS methods.

Models	Average Accuracy (%)	Average AUC	Average F-Measure
REMFFS	80.62	0.771	0.778
AREMFFS	81.67	0.784	0.797

**Table 11:** Models based on the DT classifier with the proposed AREMFFS and REMFFS methods.

Models	Average Accuracy (%)	Average AUC	Average F-Measure
REMFFS	82.75	0.699	0.813
AREMFFS	83.31	0.723	0.825

**Table 12:** Summary of the Scott–Knott rank test of the experimented FS methods on NB and DT models.

Statistical Rank	Average Accuracy		Average AUC		Average F-Measure	
	NB	DT	NB	DT	NB	DT
**1**	AREMFFS, REMFFS	AREMFFS, REMFFS	AREMFFS, REMFFS	AREMFFS	AREMFFS, REMFFS	AREMFFS, REMFFS
**2**	-	-	-	REMFFS	-	-

**Table 13:** Answers to research questions.

Research Questions	Answers
RQ1. How effective is the proposed AREMFFS method compared to baseline FFS methods?	The proposed AREMFFS outperformed individual FFS methods with statistically significant differences.
RQ2. How effective is the proposed AREMFFS method compared to existing rank aggregation-based multi-filter FS methods?	The proposed AREMFFS outperformed existing rank aggregation-based multi-filter FS methods with statistically significant differences.

## Data Availability

Not applicable.
